# Textural, Microstructural and Chemical Characterization of Ferritic Stainless Steel Affected by the Gold Dust Defect

**DOI:** 10.3390/ma16051825

**Published:** 2023-02-23

**Authors:** Beatriz Amaya Dolores, Andrés Ruiz Flores, Andrés Núñez Galindo, José Juan Calvino Gámez, Juan F. Almagro, Luc Lajaunie

**Affiliations:** 1 Laboratory & Research Section, Technical Department, Acerinox Europa S.A.U., 11379 Palmones, Spain; 2 Department of Materials Science and Metallurgical Engineering and Inorganic Chemistry, IMEYMAT, Faculty of Science, University of Cadiz, 11510 Puerto Real, Spain

**Keywords:** ferritic stainless steel, AISI 430, grains texture, electron backscatter diffraction (EBSD), microstructure, transmission electron microscopy (TEM), monochromated electron energy-loss spectroscopy (EELS), oxidations states, machine learning, k-means clustering

## Abstract

The “gold dust defect” (GDD) appears at the surface of ferritic stainless steels (FSS) and degrades their appearance. Previous research showed that this defect might be related to intergranular corrosion and that the addition of aluminium improves surface quality. However, the nature and origin of this defect are not properly understood yet. In this study, we performed detailed electron backscatter diffraction analyses and advanced monochromated electron energy-loss spectroscopy experiments combined with machine-learning analyses in order to extract a wealth of information on the GDD. Our results show that the GDD leads to strong textural, chemical, and microstructural heterogeneities. In particular, the surface of affected samples presents an α-fibre texture which is characteristic of poorly recrystallised FSS. It is associated with a specific microstructure in which elongated grains are separated from the matrix by cracks. The edges of the cracks are rich in chromium oxides and MnCr_2_O_4_ spinel. In addition, the surface of the affected samples presents a heterogeneous passive layer, in contrast with the surface of unaffected samples, which shows a thicker and continuous passive layer. The quality of the passive layer is improved with the addition of aluminium, explaining the better resistance to the GDD.

## 1. Introduction

**Figure 1:**
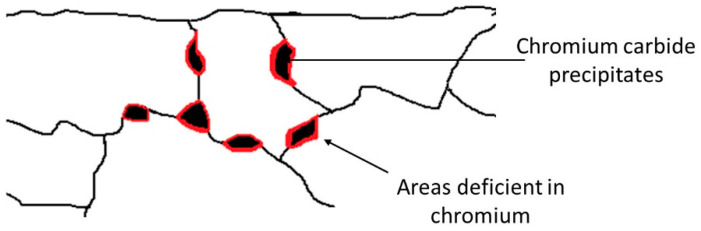
Schematic representation of the precipitation of different phases, such as chromium carbides and mixed carbides at the grain boundaries in stainless steel during sensitisation.

Ferritic stainless steels (FSS) are used today in many applications, such as cutlery, washing machine drums, automotive exhaust systems, medical and dental instruments, and in many design applications [1,2,3]. FSS present high resistance to stress corrosion cracking and low costs of production [4]. However, one of the main disadvantages of FSS is their high susceptibility to intergranular (IG) corrosion and associated defects [4]. These defects include the “gold dust” defect (GDD), which corresponds to flakes of the material that detach or semi-detach from the surface at the end of the production process. When light falls on the material, the surface roughness induced by the missing flakes gives the surface a sparkling appearance, hence the name of the GDD [5,6,7,8]. One of the main hypotheses is that this defect is related to IG corrosion. However, the nature and origin of this defect are not properly understood yet. IG corrosion usually originates from an accumulation of precipitates rich in chromium [5], which leads to a decrease in chromium at the periphery of these precipitates (Figure 1). The chromium content in these localised areas is then too low to maintain the passivity of the steel, and therefore localised sensitisation occurs, leading to IG corrosion [5,6,9,10,11].

On the other hand, previous studies have shown that corrosion resistance is closely related to the crystalline texture of ferritic stainless steel [12,13]. Correct recrystallisation of cold-rolled and annealed FSS leads to the grain orientation with (111) family planes perpendicular to the normal direction of the sheet (ND). This typical orientation is called the γ-fibre [14,15,16]. This fibre is characterised by two typical texture components in the FSS: (111)<112>, which is dominant, and (111)<110>, which is usually found in a smaller proportion. However, if the steel is not properly recrystallised, other rolling textural components may remain in the microstructure, which induces some modifications of the mechanical properties, such as ductility and elastic modulus, among others [3]. These textural components correspond mostly to the α-fibre, also called RD-fibre, because the crystals are pointing out to the rolling direction (RD) [17,18]. The most common components are (110)<110> and (001)<110>. The last component (001)<110> is related to the formation of ridges on the stainless steel surface [19,20]. On the other hand, the axes <100> parallel to ND are related to the formation of columnar grains [21]. Furthermore, it has been studied and demonstrated that stainless steel surfaces are more susceptible to corrosion when they have a higher proportion of grains with (001) orientation, while (111) and (110) orientations lead to higher corrosion resistance in the material [22]. 

As can be seen from the literature, there is a strong link between corrosion resistance and crystalline properties, such as the crystalline texture. In addition, it is known that the recrystallisation process in FSS could be improved by using grain refinements such as aluminium [23,24]. Following this analogy, the aim of this study is to focus our attention on the possible correlation between the GDD and the texture and microstructure of FSS as well as the improvement in the defect by using aluminium. Our previous work on the GDD highlighted that the addition of Al helps to decrease the damage induced by the GDD, although it was not clear how it worked [5]. The current study aims to fill this gap. To go about this, we combined several techniques such as electron backscatter diffraction (EBSD), scanning and transmission electron microscopies (STEM/TEM), as well as complementary spectroscopic techniques such as energy-dispersive X-ray spectroscopy (EDS) and monochromated electron energy-loss spectroscopy (EELS) analyses combined with machine learning analyses. 

## 2. Materials and Methods

Two samples of AISI 430 FSS were produced in the ACERINOX EUROPA S.A.U. factory, located in the Bay of Algeciras (Cádiz, Spain). The rolling stage (both hot and cold) and the subsequent annealing process were the main steps undergone by the samples. The thickness of the sheets was reduced from 200.0 mm to 3.5 mm in hot rolling at a temperature of 1000 °C, and down to 0.5 mm in the cold rolling step. Finally, the stainless steel sheets were subjected to a final annealing treatment performed in a reducing atmosphere (mixture of 75% H_2_ and 25% N_2_) at a temperature of 900 °C, which was maintained for 20 min. This annealing is called “bright annealing” because of the shiny appearance of the sheets at the end of the process. Table 1 shows the composition of the two samples as given by the factory. The main difference lies in the aluminium content, which is about 4 times higher for the sample FSS2. According to our previous study [5], this sample should thus prevent a higher concentration of GDD defects and could be used as reference sample.

### 2.1. Optical Microscopy and EBSD Characterization

Optical microscopy images were taken by using an Olympus GX71 optical microscope at 200× magnification. To reveal the cavities caused by the GDD, the tape test was applied. This test consisted of peeling off the flakes from the surface by using adhesive tape; the tape had a width of 24 nm and was supplied by Kreep Miarco. First of all, the area of interest was marked and observed by optical microscopy. Afterwards, the tape was applied and peeled off, in order to remove the flakes observed. Subsequently, the same area was studied again by optical microscopy. Finally, a statistical study was carried out to quantify the damage caused by GDD per unit area.

EBSD characterisation was performed by using a ZEISS ULTRA 55 FEG-SEM. The samples were cut in a dimension of 2 × 2 cm^2^ and were polished first in longitudinal sections by using a metallographic procedure in which sandpapers with decreasing grit sizes were used. The last step of the preparation included a final polishing with a colloidal silica suspension in order to give the surface a specular finish. The SEM used is equipped with an HKL CHANNEL 5 system from Oxford Instruments. Finally, EBSD maps were acquired at 20 kV with a working distance of 16 mm, a step size of 0.04 microns, and by using Tango and Salsa post-processing software 5.12.74.0 (Oxford Instruments, Oxford, UK). The observation plane for the analysis was ND–RD, in which ND is the normal direction (perpendicular to the sample surface), and RD is the rolling direction of the stainless steel. The FEG-SEM studies were carried out with the samples polished in longitudinal section (ND–RD plane).

### 2.2. TEM Sample Preparation and TEM Analyses

TEM lamellas were prepared by using a focused ion beam scanning electron microscope (FIB-SEM) ZEISS Crossbeam 550. It is equipped with a gallium ion gun and carbon–platinum deposition systems. The SEM image resolution was 0.7 nm, and the FIB resolution was 3 nm at 30 kV. Pt layers were deposited by the FIB to protect the lamellas from ionic damage.

Electron energy-loss spectroscopy (EELS) was carried out using an FEI Titan Cube Themis microscope operating at 200 kV. The Themis is equipped with a double Cs aberration corrector, a monochromator, an X-FEG gun, and an ultra-high resolution energy filter (Gatan Quantum ERS) that allows it to work in Dual-EELS mode. The EELS spectra were acquired with the monochromator excited and in Dual EELS mode, which allowed calibration and correction of energy instabilities by simultaneously recording the low-loss and core-loss spectra. The values of convergences and collection angles were 79.0 mrad and 33.7 mrad, respectively. EELS spectra, which were acquired for elemental quantification, were acquired with an energy dispersion of 0.5 eV/pixel, an acquisition time of 0.05 s/pixel, and an energy resolution of 1.3 eV. Most of EELS spectra, which were used for oxidation states analyses, were acquired with an energy dispersion of 0.025 eV/pixel, an acquisition time of 0.05 s/pixel, and an energy resolution of about 0.2 eV. Data EELS analyses were performed by using the Digital Micrograph software and the hyperspy package [25]. In particular, k-means clustering and white-line ratio analyses were performed by using the hyperspy package and homemade python routines. For the white-line L_3_/L_2_ ratio determination, a calculated hydrogenic ionisation cross-section was subtracted from the dataset and followed by area integration (after background and multiple-scattering removals) [26]. K-means clustering was performed by using the k-means++ algorithm [27,28]. K-means clustering is one of the most widely used unsupervised machine learning algorithms for dividing a dataset into clusters. The algorithm separates the data into clusters depending on their similarities [29]. In this case, the Cr L_3_,L_2_ lines were defined as the clustering hierarchy. For the number of clusters *k*, different metrics such as “silhouette”, “gap”, and “elbow” were used to compare the obtained results.

An FEI Talos F200X microscope (ThermoFisher, Waltham, MA, USA) operating at 200 kV was used for the STEM-EDS analyses. It is equipped with a high brightness field emission gun (X-FEG) with a resolution below 0.16 nm at 200 kV and a high-sensitivity dispersive X-ray spectroscopy detection system (Chemi-STEM G2 technology) integrating 4 Silicon Drift Detectors (SDD) around the sample, which have symmetric design and are windowless and shutter-protected. The samples were analysed in scanning mode (STEM) and with a high-angle annular dark field detector (HAADF). EDS quantification was performed by using the Velox software version 3.4.0 (ThermoFisher, Waltham, MA, USA) with parabolic background and Brown–Powell ionisation cross-section models [30]. The EDS resolution of the microscope is equal to 136 eV in the Mn-K-α line. The Cu-K lines came mostly from the TEM sample holder and were deconvoluted prior to quantification. Due to the proximity between the Cr-L and O-K lines, the Cr-K lines were used for chromium quantification.

## 3. Results

### 3.1. Surface Characterisation by Optical Microscopy

**Figure 2:**
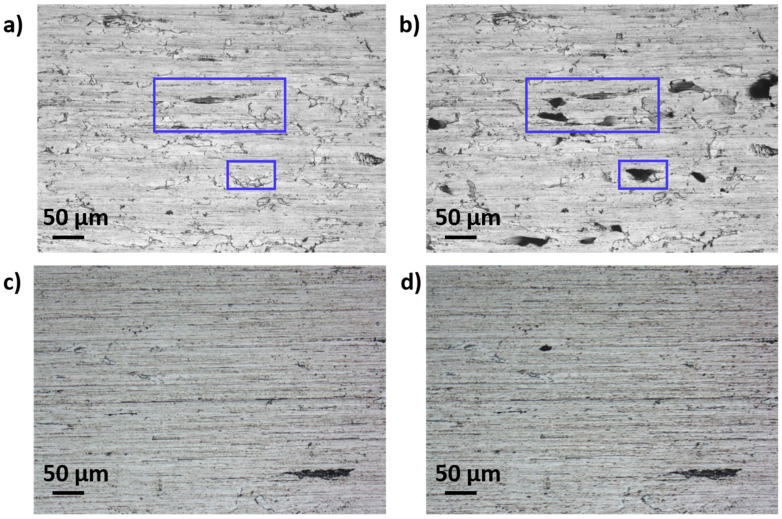
Optical microscopy images of the surface of the FSS1 sample (**a**) before and (**b**) after the tape test. The two blue rectangles indicate flakes that were detached during the tape test. Optical microscopy images of the surface of the FSS2 sample (**c**) before and (**d**) after the tape test.

Figure 2 shows the optical microscopy images of the surface of the samples, before and after the tape test. In particular, Figure 2a,b show the surface of the FSS1 sample before and after the tape test, respectively. It can be observed that many flakes are detached from the surface after the tape test, leaving cavities at the surface of the material. On the other hand, for the FSS2 sample, there is hardly any difference before and after the test (Figure 2c,d). Statistical analyses of the images show about 6.0% of the surface is affected by the GDD for the FSS1 sample, whereas only 0.7% of the surface of the FSS2 sample is affected by the GDD. It confirms that a higher Al content leads to an improvement in the quality surface of the samples [5].

### 3.2. Textural Analyses

**Figure 3:**
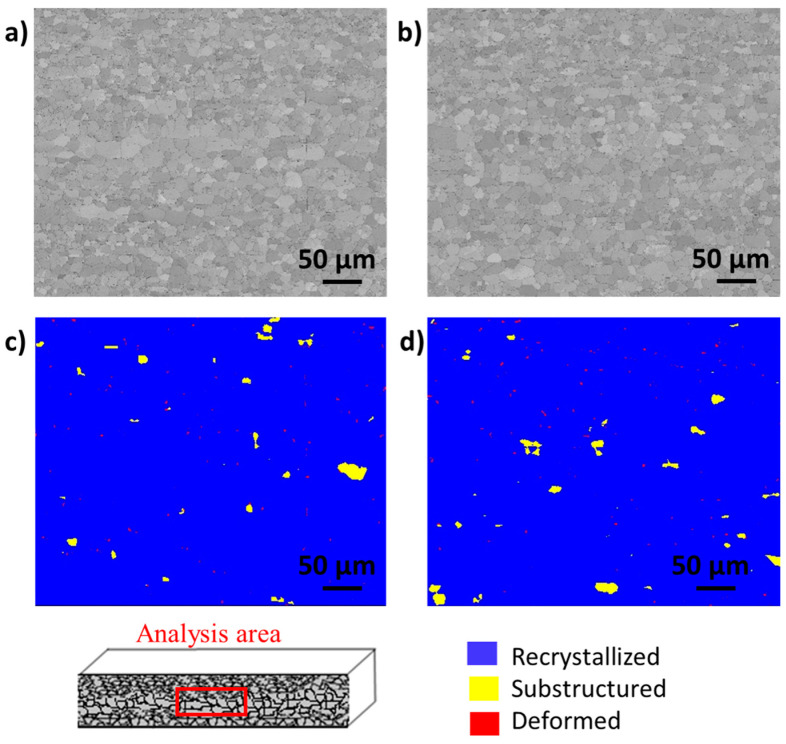
(**a**) Band contrast (BC) map acquired on the FSS1 sample, (**b**) BC map acquired on the FSS2 sample, (**c**) DefRex map acquired on the FSS1 sample, (**d**) DefRex map acquired on the FSS2 sample. All the data were acquired at the centre of the sheets prepared in cross-section geometry.

A detailed electron backscatter diffraction (EBSD) analysis of the samples prepared in cross-section geometry was performed on both samples. In addition, for each sample, the analyses were performed at the centre of the stainless steel sheets and on the surface of the sheets. Figure 3 shows the band contrast (BC) and deformation–recrystallisation maps (DefRex) of samples FSS1 and FSS2. The BC maps describe the average intensity of the Kikuchi bands in relation to the overall intensity and are particularly useful for highlighting the grain boundaries. The recrystallisation maps are based on the degree of disorientation between the grains. For grains with a disorientation lower than one degree, the software identifies them as deformed subgrains (highlighted in red on the map). If the disorientation is between 1 and 7.5°, the grains are displayed as partially recrystallised (highlighted in yellow on the map). Finally, if the disorientation is above 7.5°, they are displayed as well-defined grains and are highlighted in blue on the map. These analyses were performed first at the centre of the cross-section of the samples (cf. legend of Figure 3). It can be seen that for both samples, the recrystallisation is almost complete: 98% of the grain is recrystallised at the centre of both samples. Therefore, far from the surface, there is almost no difference between the samples that are affected and unaffected by the GDD.

**Figure 4:**
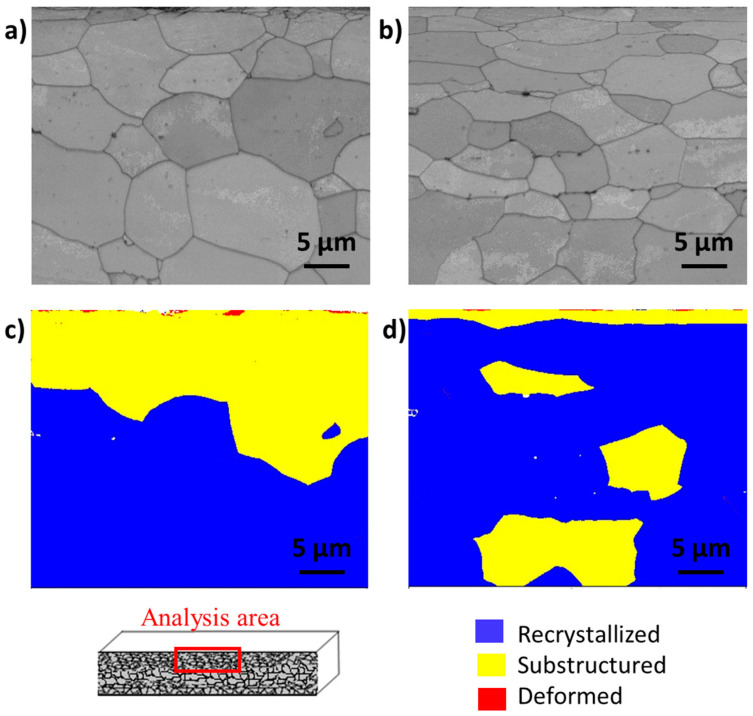
(**a**) BC map acquired on the FSS1 sample, (**b**) BC map acquired on the FSS2 sample, (**c**) DefRex map acquired on the FSS1 sample, (**d**) DefRex map acquired on the FSS2 sample. All the data were acquired at the surface of the sheets prepared in cross-section geometry.

Figure 4 shows a similar analysis performed on both samples prepared in cross-section geometry, but in this case, the analyses were performed close to the surface of the sheets. The probed areas were taken just below a flake affected by the GDD. As can be seen in Figure 4a,b, both samples show a large grain size distribution. The smallest grain area is about 0.02 µm^2^, and the largest is about 230 µm^2^. The recrystallisation of FSS1 is considerably poorer close to the surface (Figure 4c) than for FSS2 (Figure 4d). In particular, only 58.8% of the grains of FFS1 are completely recrystallized, 40.9% form substructures, and 0.3% are deformed. Moreover, the affected grains are mostly present close to the surface, down to about 20 µm. In the case of FSS2, 77.9% of the grains are completely recrystallised, 22.0% form substructures, and 0.1% are deformed. Most of the affected grains with a disorientation lower than 7.5° are closest to the surface, down to five microns. However, some grains located at a greater depth are also not properly recrystallised.

**Figure 5:**
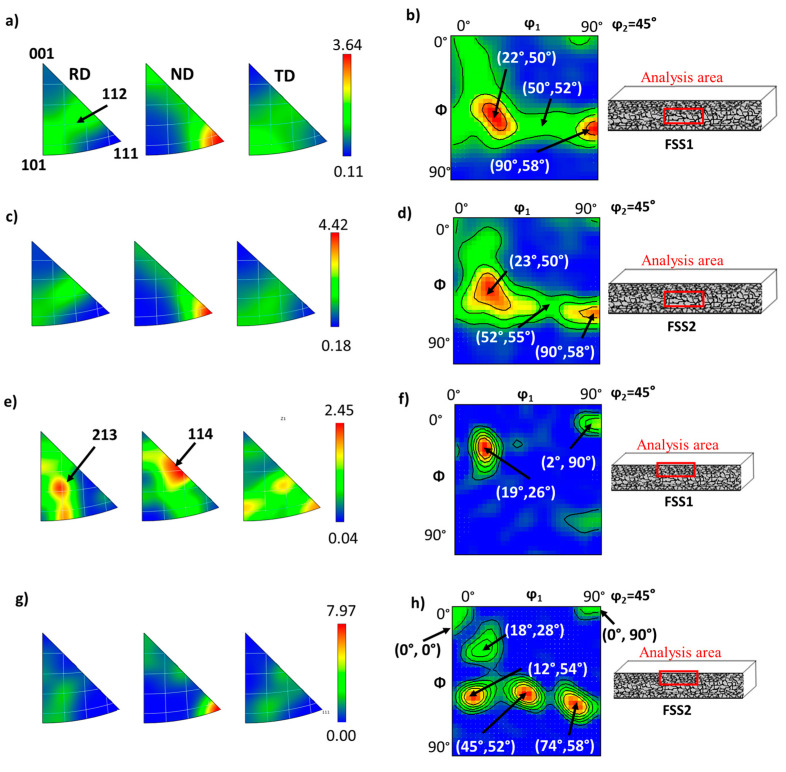
(**a**) Inverse pole figures (IPF) acquired on the FSS1 sample at the centre of the sheet, (**b**) corresponding orientation density function (ODF) map, (**c**) IPF acquired on the FSS2 sample at the centre of the sheet, (**d**) corresponding ODF map, (**e**) IPF acquired on the FSS1 sample at the surface of the sheet, (**f**) corresponding ODF map, (**g**) IPF acquired on the FSS2 sample at the surface of the sheet, and (**h**) corresponding ODF map.

Figure 5 shows the inverse pole figures (IPF) and orientation density function (ODF) maps obtained on both samples and acquired on two different areas of the samples prepared in cross-section geometry: (i) at the surface and (ii) at the centre of the sheets. The IPF maps represent the relative distribution of the crystal axes with respect to the rolling direction (RD), the normal direction (ND), and the transverse direction (TD) of the sample. 

Figure 5a–d show the ODF and IPF maps acquired on the two samples at the centre of the sheets. The IPF maps (Figure 5a,c) show that for both samples, most of the crystals present their axes <111> parallel to ND, confirming a correct recrystallisation of the grains. Specifically, the texture is bimodal and is dominated in the majority by the (111)<112> component and by the (111)<110> component to a lower extent. These components belong to the γ-fibre, which is the expected texture at the end of a proper recrystallisation process [13]. In Figure 5b,d, the ODF maps clearly confirm that the preferential orientations at the centre of the samples correspond to the γ-fibre. These orientations are highlighted at about (90°, 58°, 45°) and (50°, 55°, 45°) positions which correspond to (111)<112> and (111)<110> orientations, respectively. Finally, there is an intense node at (23°, 50°, 45°), which corresponds to a mix of (111)<1$\bar{2}$1> and (334)<4$\bar{8}$3>. All of these orientations correspond to the same fibre confirming optimal recrystallisation. This can also be verified in Figure S1 (located in the Supplementary Materials section), which shows the possible orientations for an ideal BCC stainless steel. 

Figure 5e–h show the ODF and IPF maps acquired on the two samples at the surface of the sheets. For sample FSS1, a clear difference is highlighted between the IPF and ODF maps acquired at the centre and at the surface of the sheets. In particular, the texture observed in the IPF maps of the FSS1 sample at the surface (Figure 5e) presents the axis <114> parallel to ND, instead of the axis <111> as observed for the same sample at the centre of the sheet. In addition, the ODF map (Figure 5f) presents a strong component at (19°, 26°, 45°), which corresponds to (115)<110>, and a lower intense node at (2°, 90°, 45°), which corresponds to (001)<110> orientation. These orientations belong to the α-fibre confirming that the rolling texture predominates close to the surface of this sample. It confirms thus that the grains at the surface are not properly recrystallised for the FSS1 sample. On the other hand, Figure 5g shows that the texture at the surface of the FSS2 sample has the direction <111> parallel to ND, which is similar to the analysis performed at the centre of the sheet. In addition, in the corresponding ODF map (Figure 5h), it can be clearly seen that the rolling orientations of the γ-fibre dominate the texture of the surface of the FSS2 sample, the highest intensity nodes corresponding to the γ-fibre at (334)<4$\bar{8}$3>, (111)<110> and (111)<112>. Although other nodes are present with a lowest intensity at (0°, 0°, 45°), (0°, 90°, 45°), and (18°, 28°, 45°), corresponding to (001)<1$\bar{1}$0>, (001)<$\bar{1}\bar{1}$0>, and (115)<110> orientations. 

As can be seen from the textural analysis, the behaviour of both samples at the centre of the plates is similar and corresponds to proper recrystallised ferritic stainless steels [31]. However, close to the surface, the behaviour of FSS1 is strongly affected by the GDD and presents the characteristic texture of improperly recrystallised ferritic stainless steel. On the other hand, the surface of the sample FSS2 (richer in Al than FSS1) shows mainly a texture characteristic of a proper recrystallisation process. This confirms that the recrystallisation process was complete for this sample.

### 3.3. EELS Analysis and Machine-Learning

**Figure 6:**
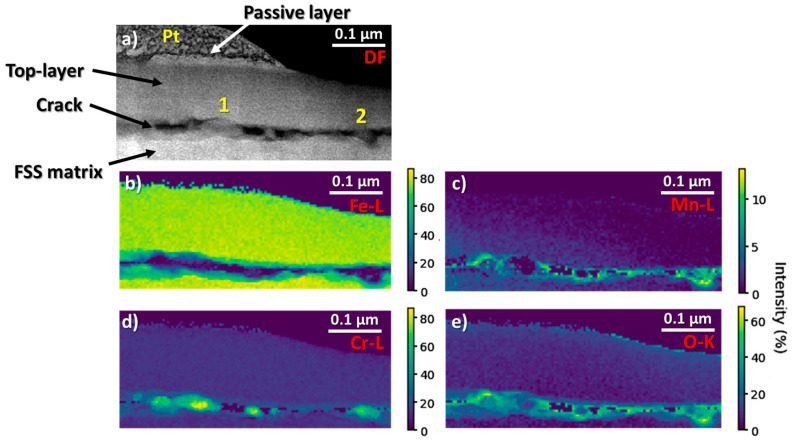
(**a**) Dark-field micrograph of the surface of a lamella of the FSS1 sample acquired simultaneously as the EELS data. Quantification elemental maps (in at.%) for the (**b**) Fe, (**c**) Mn, (**d**) Cr, and (**e**) O elements.

EELS analyses (Figure 6) were carried out to reveal and quantify any chemical heterogeneities at the surface of FSS1, this sample presenting poorer recrystallisation according to the EBSD results. It should be noted that the corresponding lamella was prepared in cross-section geometry just underneath a flake affected by the GDD. At the surface of the sample, a top layer with a thickness of about 0.25 µm is clearly observed, and it is separated from the FSS matrix by a crack (Figure 6a). This kind of microstructure is characteristic of the GDD [5]. The corresponding monochromated EELS spectra extracted from the dataset are displayed in Figure S2, located in the Supplementary Materials, and are commented on in more detail. Briefly, differences in the Cr local chemical environment can be observed between the top layer and the edges of the crack as well as chemical heterogeneities in the compounds situated at the edges of the crack. The corresponding elemental maps were extracted and are shown in Figure 6b–e. It can be seen that the Fe distribution is homogeneous in both the top layer and the FSS matrix, and is equal to 76.7 ± 1.4 at.%. On the other hand, a strong decrease in the Fe content is observed at the edge of the crack, reaching a value of 49.1 ± 4.7 at.%. It can also be observed that the chemical composition of the crack is complex and heterogeneous. Two different kinds of areas can be highlighted along the crack. In this case, two representative areas of these zones were marked as 1 and 2 (mentioned above): (1) area without Mn, which corresponds to the area with high Cr content (78.7 ± 6.1 at.%) and an O content equal to 20.0 ± 1.2 at.% and (2) area, which are richer in Mn (12.5 ± 0.7 at.%), with Cr and O contents equal to 28.1 ± 1.2 and 56.4 ± 2.5 at.%, respectively.

**Figure 7:**
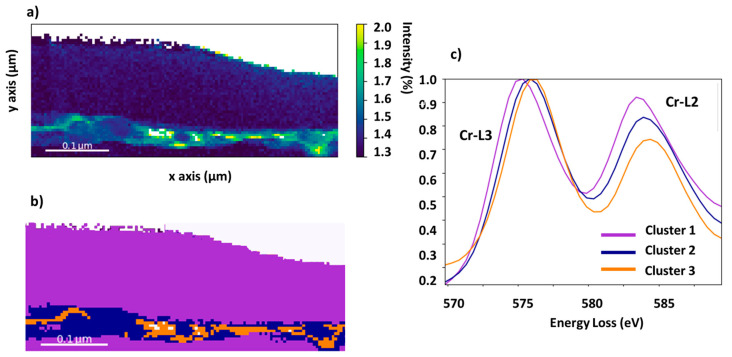
(**a**) Cr-L_3_/L_2_ ratio intensity map. (**b**) K-means cluster map using the Cr-L_2,3_ edges and (**c**) the corresponding EELS signals associated with each cluster.

To obtain more details on these heterogeneities, the Cr local environment was quantified by calculating the Cr-L_3_/L_2_ ratio as well as by using a machine-learning-based approach (Figure 7). Figure 7a shows the Cr-L_3_/L_2_ ratio intensity map as calculated from the monochromated EELS dataset. It can be observed that the top layer has a Cr-L_3_/L_2_ ratio of 1.3 ± 0.1, which is in good agreement with the values obtained for Cr^0^ [32,33,34]. In the case of the compounds situated at the edges of the cracks, the Cr-L_3_/L_2_ ratio seems to follow a bimodal distribution with two main values centred around 1.7 ± 0.1 and 2.1 ± 0.2. The value of 1.7 is found in the areas where there is no Mn content and is in good agreement with the value reported for Cr_2_O_3_ [32,34,35]. In addition, values of 2.1 for the Cr-L_3_/L_2_ ratio are also frequently observed at the edges of the crack, coinciding with the presence of Mn. The quantification performed in these areas yields values of Cr/O = 0.5, Mn/O = 0.2, and Cr/Mn = 2.3, which is consistent with the formation of the spinel MnCr_2_O_4_. It should be noted that the formation of this spinel was already reported in FSS at high temperatures [36]. All these results are in good agreement with the variation of EELS fines structures as discussed in the Supplementary Materials (cf. Figure S2 in the Supplementary Materials). It should be noted that the local chemical environment of Mn does not vary over the probed area. Moreover, by implementing k-means clustering, only one cluster (located in the crack) is obtained as a result. These results can be seen in Figure S3, located in the Supplementary Materials. It is important to note that the proper evaluation of the Mn-L_3_/L_2_ ratio is delicate due to the low Mn content.

To check the bimodal distribution of the Cr L_2,3_ EELS fine structures, the EELS dataset was clustered by using only the energy range corresponding to the Cr-L_2,3_ edges. For this purpose, the “k-means++” algorithm was used [27], and the number of clusters was automatically selected by using the silhouette metric [37], which showed the presence of three different clusters (cf. Figure S4a in the Supplementary Materials). It should be noted that other metrics also indicate the presence of three clusters (cf. Figure S4b,c in the Supplementary Materials). The corresponding k-means clustering map and the EELS signal associated with each cluster are shown in Figure 7b,c, respectively. It can be seen that there is an excellent correlation between the cluster map, the Cr-L_3_/L_2_ ratio map, and the elemental maps. In particular, cluster 1 (highlighted in purple in Figure 7b) corresponds to both the top layer and the matrix, which present the lowest value of the Cr-L_3_/L_2_ ratio. Cluster 2 (highlighted in blue in Figure 7b) corresponds to the edges of the crack with the highest Cr content and intermediate values of the Cr-L_3_/L_2_ ratio. Finally, cluster 3 (highlighted in orange in Figure 7b) corresponds to the edges of the crack with the highest Mn content and the highest values of the Cr-L_3_/L_2_ ratio. All these results show the presence of at least two compounds at the edge of the crack: one rich in Cr and O, and another compound rich in Cr, Mn, and O.

### 3.4. STEM Analyses

**Figure 8:**
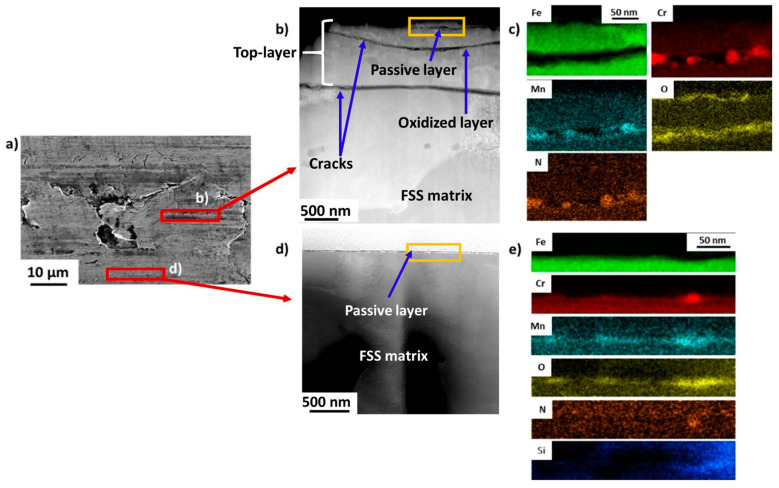
STEM analyses acquired on the FSS1 sample. (**a**) SEM micrograph. The two red rectangles highlight the areas which were used to prepare the TEM lamellas. (**b**) STEM-HAADF micrograph of an area affected by the GDD. The rectangle highlights the area used to perform the EDS analyses. (**c**) Corresponding EDS chemical maps. (**d**) STEM-HAADF micrograph of an area unaffected by the GDD. The rectangle highlights the area used to perform the EDS analyses. (**e**) Corresponding EDS chemical maps.

Figure 8 shows the TEM analyses performed on the FSS1 sample. For each sample, two different TEM lamellas were prepared by FIB: (i) one lamella prepared from an area affected by the GDD and (ii) one prepared from an unaffected area.

Figure 8a shows an SEM micrograph acquired on the FSS1 sample. The presence of flakes at the surface, which are characteristics of the GDD, can be highlighted [5]. The dimensions of the flakes are about 60 × 25 µm. Figure 8b,d show the STEM-HAADF micrographs acquired on the affected and unaffected areas, respectively. It can be observed that the microstructures of the two TEM lamellas are completely different. 

For the lamella prepared directly below an affected flake, a top-layer microstructure is observed, as expected. In this case, it has a thickness of about 1.5 microns and is separated from the matrix by two cracks (highlighted in Figure 8b). The corresponding EDS chemical maps of this sample at low magnification are shown in Figure S5, located in the Supplementary Materials. It can be seen that the top layer is composed of an accumulation of grains rich in Cr and deficient in iron in general. The presence of O is also highlighted near the cracks, and precipitates rich in Cr, N, and V are also observed. 

On the other hand, the TEM lamella, which was prepared from an unaffected surface, does not show the presence of a top-layer microstructure (Figure 8d). The surface in this area is flat, and the presence of cracks cannot be highlighted. Figure 8c,e show the EDS analyses which were performed at the extreme surface of both lamellas. In the case of the TEM prepared on the GDD flake (Figure 8c), the typical passive layer that naturally forms on FSS is not observed on the surface [38]. Steels are stainless (with at least 10.5 wt.% Cr) due to the presence of an oxidised layer that is spontaneously generated on the surface of the steel. This layer is called the passive layer, and the process of its formation is the passivation process. Its thickness is usually in the order of a few nanometres, and it protects the rest of the material from external agents. Commonly, in a conventional AISI 430 FSS, this passive layer is rich in chromium oxides [38]. The corresponding EDS maps show the presence of a discontinuous oxidised layer, but other elements are hardly highlighted. So, the passive layer seems to have weakened due to the GDD. This is typically seen in FSS samples affected by IG corrosion [8]. However, the edges of a crack located 50 nm below the surface show the presence of a discontinuous and heterogeneous oxidised layer with the following composition: Cr: 33.1 ± 1.5 at.%, Mn: 8.5 ± 1.4 at.%, N: 18 ± 1.7 at.% and O: 40.4 ± 5.3 at.%. It has an uneven distribution of Cr and N and a thickness of about 12 nm. In the case of the TEM flake prepared far from the flake (Figure 8e), a passive layer is observed at the surface of the sample. It is mainly rich in Cr (21.2 ± 3.2 at.%), Mn (7.8 ± 1.2 at.%), O (53.0 ± 5.4 at.%), and N (18.0 ± 2.1 at.%). This passive layer is also heterogeneous and discontinuous, and has a thickness of about 12 nm. Finally, in Figure S6a–d, located in the Supplementary Materials, some precipitates which are rich in Cr, N, V, and C and deficient in Fe are also observed.

**Figure 9:**
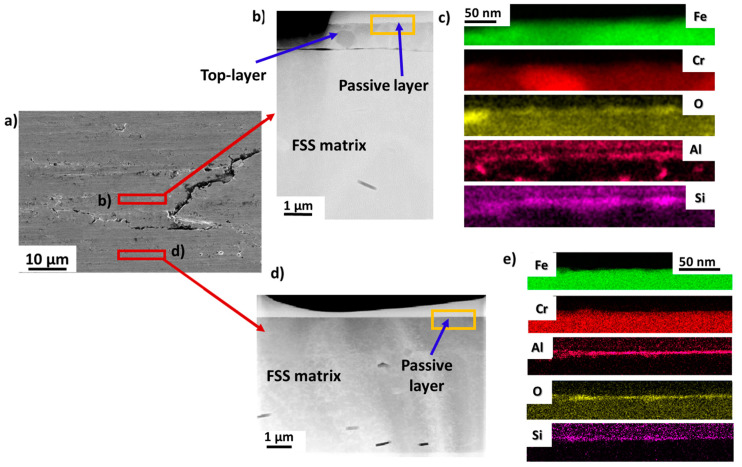
STEM analyses acquired on the FSS2 sample. (**a**) SEM micrograph. The two red rectangles highlight the areas which were used to prepare the TEM lamellas. (**b**) STEM-HAADF micrograph of an area affected by the GDD. The rectangle highlights the area used to perform the EDS analyses. (**c**) Corresponding EDS chemical maps. (**d**) STEM-HAADF micrograph of an area unaffected by the GDD. The rectangle highlights the area used to perform the EDS analyses. (**e**) Corresponding EDS chemical maps.

Figure 9 shows the same analyses performed on the FSS2 sample. A flake characteristic of the GDD can also be observed at the surface of the sample in the top-view geometry (Figure 9a). The dimensions of the flake are around 50 × 20 µm. Figure 9b,d show the STEM-HAADF micrographs acquired on the affected and unaffected areas, respectively. The same microstructures as the other sample are observed. The lamella prepared on the affected area shows the presence of a top-layer microstructure, while the other one shows a flat and perfect surface. Concerning the affected area, the general quality of the FSS2 sample is better than that of the FSS1 sample: both the interfaces between the surface and the top layer and between the top layer and the matrix are flat, and no cracks are highlighted inside the top layer. The thickness of the top layer decreased with respect to the FSS1 sample and is equal to 900 nm. It confirms that the FSS2 sample is less affected by the GDD than the FSS1 sample. In Figure S7 in Supplementary Materials, it can be observed that the top layer presents an accumulation of grains rich in Cr and Al. The EDS analyses performed at the surface of both lamellas (Figure 9c,e) clearly highlight the presence of a passive layer at the surface, even in the area affected by the GDD. For both lamellas, the thickness of the passive layer is about 18 nm, and the passive layer is more homogeneous and continuous with respect to the FSS1 sample. Its chemical composition is also different from the passive layer of the FSS1 sample and is rich in O (76.5 ± 2.7 at.%), Al (16.5 ± 2.7 at.%), and Si (7.4 ± 1.3 at.%). The presence of Mn is not observed for this sample. Finally, in Figure S8a–d, located in the Supplementary Materials, the presence of precipitates that are rich in Al and N (instead of Cr, N, C, and V as for the FSS1 sample) is highlighted. 

## 4. Conclusions

In this work, we studied two ferritic stainless steels; one is strongly affected by the gold dust defect (GDD), while the surface of the other one remains mostly unaffected. In particular, we have shown that the density of defects observed at the surface is decreased by a factor of 10 upon the addition of Al. The EBSD analyses showed that the most affected sample presents a strong textural heterogeneity between the surface and the centre of the stainless steel sheet: the texture of the surface being characteristics of an improper recrystallised FSS while the centre of the sheet presents a γ-fibre texture as expected at the end of a proper recrystallisation process. On the other hand, both the surface and the centre of the sample richer in Al present a proper γ-fibre texture. By combining FIB sample preparation and STEM analyses, we showed that the GDD presents a characteristic microstructure in which elongated grains are separated from the matrix by a series of cracks. In particular, EELS analyses showed that the edges of the cracks are rich in chromium oxides and MnCr_2_O_4_ spinel. On the other hand, the unaffected areas present a flat and crack-free surface. Furthermore, we have shown that the Al-rich sample presents a continuous and homogeneous passivation layer, whereas the affected sample presents a defective passivation layer. All these results show that showed that (i) the GDD is linked to an improper recrystallisation process, (ii) that the addition of Al is able to overcome an insufficient annealing because Al acts as a grain refiner, and that (iii) Al improves the quality of the passive layer, which allows limiting the damages induced by intergranular corrosion and thus by the gold dust defect.

## Figures and Tables

**Table 1:** Chemical composition of the AISI 430 FSS samples.

	Samples	
Element at.%.	FSS1 ^1^	FSS2 ^2^
C	0.37	0.33
Cr	17.58	17.40
Mn	0.48	0.51
Si	0.98	1.05
Al	0.15	0.60
N	0.34	0.30
Fe	80.11	79.79

^1, 2^ Sample affected and unaffected by the GDD, respectively.

## Data Availability

The data presented in this study are available upon reasonable request to the corresponding authors.

## Supplementary Materials

The following supporting information can be downloaded at https://www.mdpi.com/article/10.3390/ma16051825/s1. Figure S1: Orientation distribution function, Figure S2: Cross-section of the FSS1 sample prepared by FIB in an area affected by the GDD, Figure S3: Cross-sections of the FSS1 sample prepared by FIB, Figure S4: Cross-section of FSS2 sample prepared by FIB in an area affected by the GDD, Figure S5: Cross-sections of FSS2 prepared by the FIB, Figure S6: EELS spectra, Figure S7: Intensity map of the Mn-L3/Mn-L2 ratio, and Figure S8: Cluster metrics.
