# Supplementary material for: Textural, Microstructural and Chemical Characterization of Ferritic Stainless Steel Affected by the Gold Dust Defect
Source: Materials (Basel). 2023 Feb 23;16(5):1825. doi: 10.3390/ma16051825 (PMC10004007; doi:10.3390/ma16051825)
Supplement: Supplementary file 1 [file materials-16-01825-s001.zip › materials-2191632-supplementary.docx]

**Supplementary Materials for:**

**Textural, Microstructural and Chemical Characterization of Ferritic Stainless Steel Affected by the Gold Dust Defect**

Beatriz Amaya Dolores^1^, Lidia Chinchilla^2^, Andrés Ruiz Flores^1^, Andrés Núñez Galindo^1^, José Juan Calvino Gámez^2^, Juan F. Almagro^1,^*, Luc Lajaunie^2,^*

^1^Laboratory & Research Section, Technical Department, Acerinox Europa S.A.U., 11379 Palmones, Spain

^2^Department of Materials Science and Metallurgical Engineering and Inorganic Chemistry. IMEYMAT. Faculty of Science. University of Cadiz, 11510 Puerto Real, Spain.

* Corresponding authors:

[luc.lajaunie@uca.es](mailto:luc.lajaunie@uca.es)

[juan.almagro@acerinox.com](mailto:juan.almagro@acerinox.com)

| 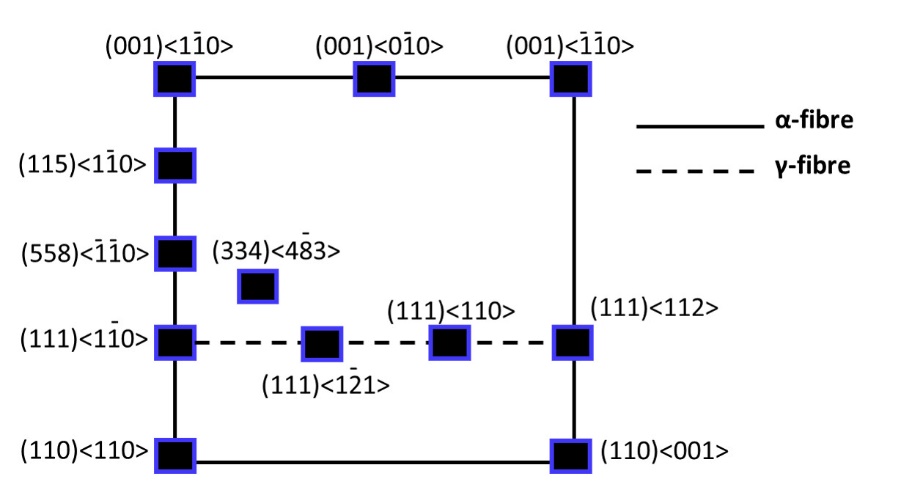 |
| --- |
| **Figure S1.** Orientation distribution function (ODF) with ϕ_2_=45° of an annealed FSS 430 representing the ideal orientations and fibres. |

Figure S1 shows the possible orientations and the corresponding fibres texture in a typical BCC stainless steel. The global texture of BCC steels can be described as by a set of different fibres mainly constituted by the α-fibre and the γ-fibre [14,15]. The α-fibre is promoted in the initial process of fabrication. It shows orientations with a common crystalline axis parallel <110> to the direction of the rolling process (RD). On the other hand, the γ-fibre which is promoted after the recrystallization process and shows orientations with <111> parallel to the normal direction (ND) that is perpendicular to the sample surface. Textural components such as the (111) <1-21> and (111) <112> belong to the γ-fibre. A FSS that is well recrystallized after the final annealing process should present a texture consisting mainly of the γ-fibres [21].

| 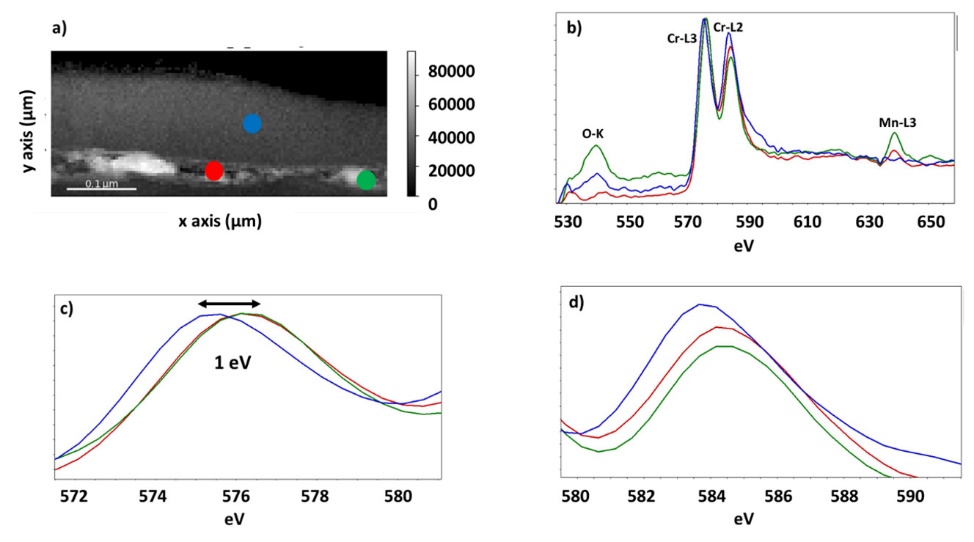 |
| --- |
| **Figure S2. a)** Dark-field image acquired simultaneously as the EELS dataset. The blue, red and green circles highlight the area used to extract the blue, red and green spectra shown in the other sub-figures, **b)** spectra from the areas highlighted in a) showing the O-K, Cr-L_23_ and Mn-L_23_ edges, **c)** Magnified area of Cr-L_3_ edge and **d)** Magnified area of Cr-L_2_ edge. |

Figure S2 shows the monochromated EELS analysis performed on the top-layer and at the interface top-layer/matrix of the FSS1 sample. Figure S2b shows clear differences between the fines structures of the O-K, Cr-L_2,3_ and Mn-L_2,3_ edges which were acquired on the top-layer and on the edges of the cracks (Figure S2b-d). In particular, between the top-layer (blue) and the edges of the crack (red and green), the position of the Cr-L_3_ lines is shifted to higher energy by 1 eV. In addition, when the Cr-L_3_ intensity is normalized, the intensity of the Cr-L_2_ line acquired on the edges of the cracks is decreased. Between the two spectra acquired at the edges of the cracks, variations in Cr-L_2_ intensities as well as O-K and Mn-L_2,3_ fine structures can be highlighted. All these results show differences in the Cr local chemical environment between the top-layer and the edges of the crack as well as chemical heterogeneities of the compounds situated at the edges of the crack.

| 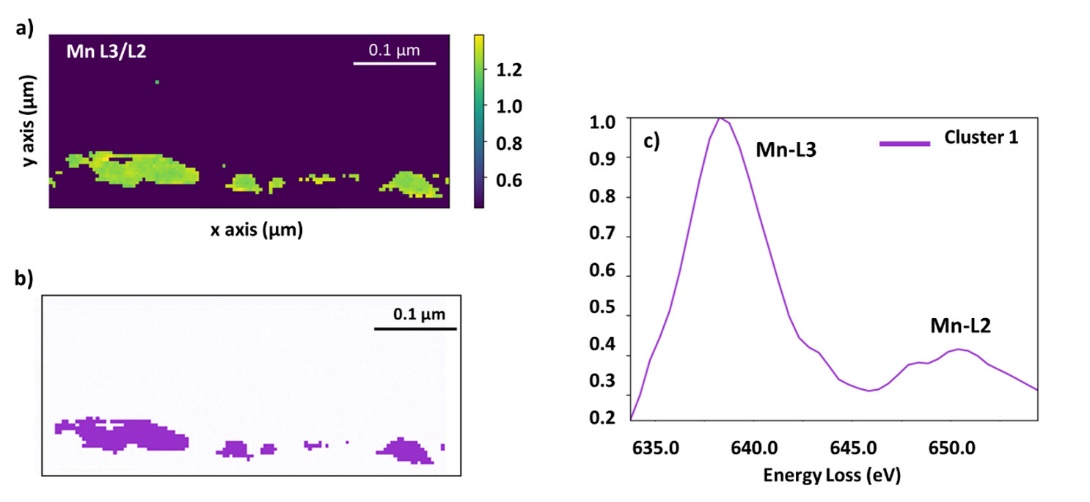 |
| --- |
| **Figure S3. a)** Intensity map of the Mn-L_3_/Mn-L_2_ ratio, **b)** KMeans Clustering map using Mn-L_3_ and Mn-L_2_ lines and **c)** Mn-L_3_ and Mn-L_2_ signal of the cluster. |

| 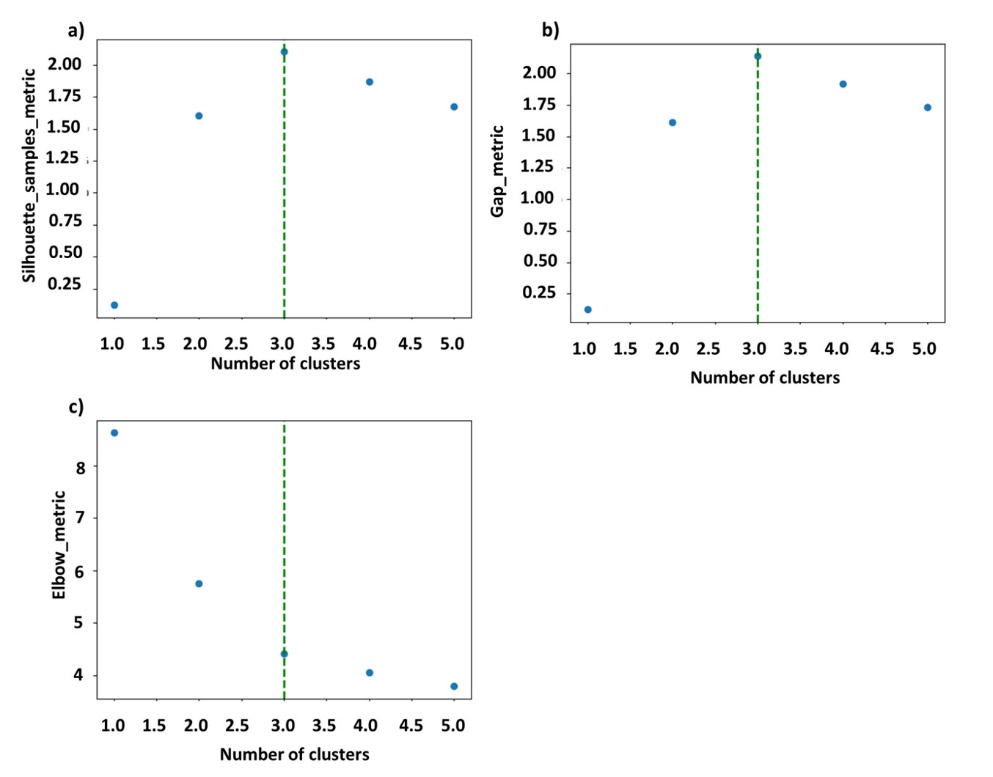 |
| --- |
| **Figure S4.** Determination of the number of clusters by using the: **a)** Silhouette, **b)** Gap and **c)** Elbow metrics. |

| 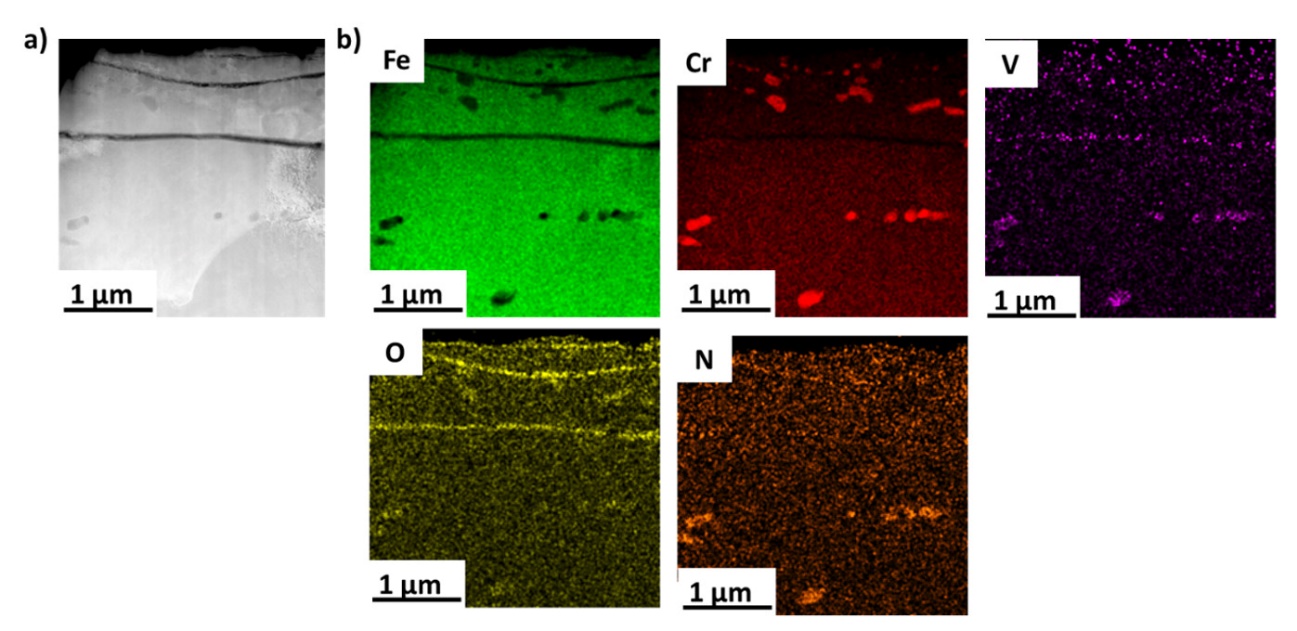 |
| --- |
| **Figure S5.** Cross section of the FSS1 sample prepared by FIB in an area affected by the GDD. **a)** STEM-HAADF image, **b)** corresponding EDS chemical maps. |

| 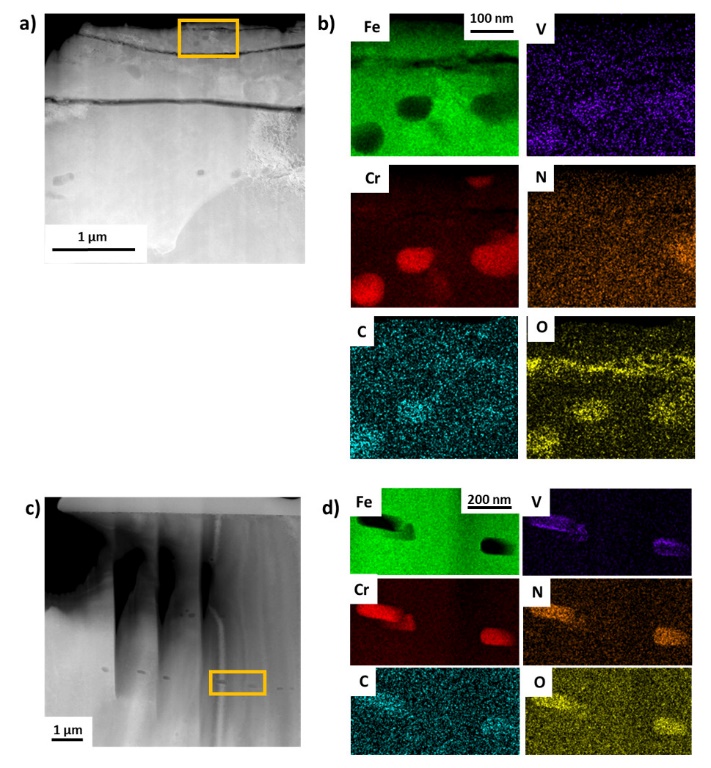 |
| --- |
| **Figure S6.** Cross sections of the FSS1 sample prepared by FIB **a)** STEM-HAADF image acquired in an area affected by the GDD. The rectangle highlights the area used for the EDS analyses. **b)** EDS chemical maps of the rectangle highlighted in the Figure S3a. **c)** STEM-HAADF image acquired in area outside of the GDD. The rectangle highlights the area used for the EDS analyses and **d)** EDS chemical maps of the rectangle highlighted in the Figure S3c. |
| 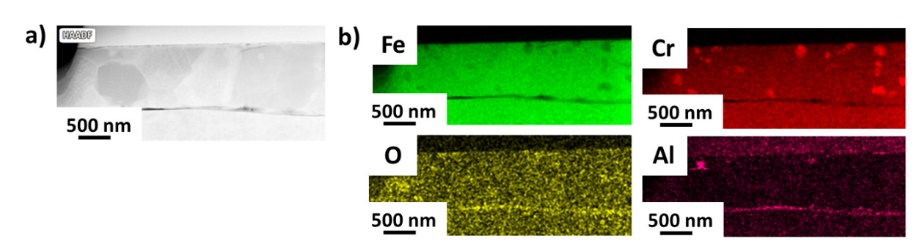 |
| **Figure S7. a)** Cross section of FSS2 sample prepared by FIB in an area affected by the GDD and **b)** The corresponding EDS chemical maps. |

| 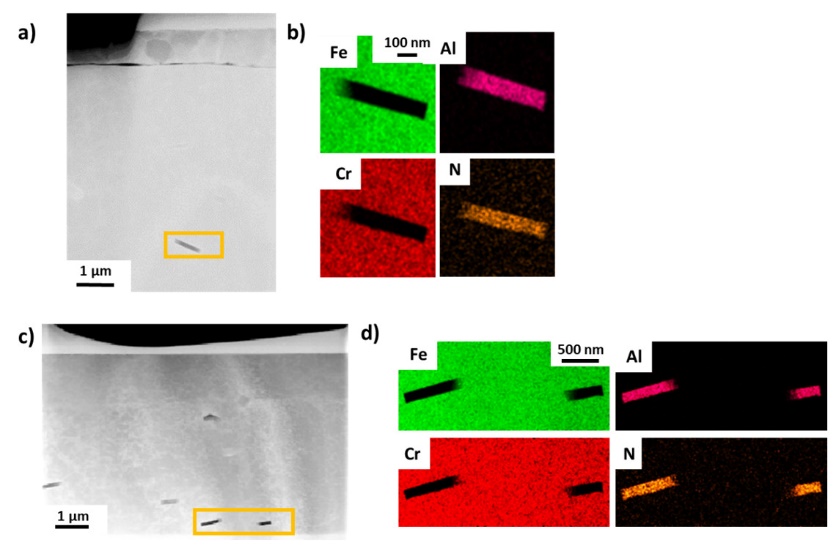 |
| --- |
| **Figure S8. Cross sections of FSS2 prepared by the FIB:** **a)** In area affected by the GDD. The rectangle highlights the precipitates of the lamella. **b)** The EDS chemical maps of the rectangle highlighted in the Figure S5a. **c)** In area outside of the GDD. The rectangle highlights the precipitates of the lamella as well, and **d)** The EDS chemical maps of the rectangle highlighted in the Figure S5c. |
